# Intermittent Glucocorticoid Pulse Combined With Mycophenolate Mofetil in Juvenile Dermatomyositis

**DOI:** 10.1001/jamadermatol.2025.4483

**Published:** 2025-11-19

**Authors:** Lishuang Guo, Jinxiang Liu, Mengjiao Xin, Guochen Yu, Chenyu Zhu, Xinyuan Zhao, Sirui Yang

**Affiliations:** 1 Department of Pediatric Rheumatology, Immunology & Allergy, Children’s Medical Center, the First Hospital of Jilin University, Changchun, China; 2 The Child Health Clinical Research Center of Jilin Province, Changchun, China

## Abstract

This cohort study reports the long-term clinical outcomes of the use of intermittent intravenous methylprednisolone pulse therapy combined with mycophenolate mofetil for patients with juvenile dermatomyositis.

Oral glucocorticoids (GCs) with immunosuppressants remain first-line treatment for patients with juvenile dermatomyositis (JDM). Intermittent intravenous methylprednisolone pulse (IVMP) therapy may offer advantages over daily high-dose oral GCs^1,2^ and has been used in autoimmune diseases, including JDM.^3^ Mycophenolate mofetil (MMF) is an alternative to methotrexate for JDM.^4,5^ For over a decade, our center has adopted intermittent IVMP plus MMF as first-line therapy. This study presents long-term clinical outcomes.

## Methods

Twenty-eight patients with JDM from May 1, 2014, to May 31, 2025, who met the 2006 Bohan/Peter criteria or 2017 EULAR/ACR classification criteria, with 4 or more years of follow-up, were analyzed. All received 7 to 9 courses of IVMP (20-30 mg/kg/d for 3 consecutive days per course) (eFigure in Supplement 1) plus MMF (500-600 mg/m^2^, twice daily). Hydroxychloroquine was added for skin disease activity scores (DASs) of 5 points or more (61% [17 of 28]); intravenous immunoglobulin was added for poor muscle strength or rash after 3 to 4 IVMP courses or severe initial symptoms (32% [9 of 28]). All received vitamin D (800 IU) and calcium (600 mg) daily. No other immunosuppressants or targeted drugs were used. Clinical data collected included Childhood Myositis Assessment Scale (CMAS) scores, the validated 20-point DAS, height and body mass index *z* scores, bone mineral density (BMD) *z* scores, laboratory parameters, and adverse events. The ethics committee of the First Hospital of Jilin University approved the study with a waiver of informed consent because data were deidentified. The STROBE reporting guideline was followed.

Data were presented as frequencies (percentages) or median (IQR) values. Generalized estimating equations and Wilcoxon signed rank tests were used for analyses. Missing data were imputed with mean values from adjacent visits. A 2-sided *P* < .05 was considered statistically significant (eMethods in Supplement 1).

## Results

Of 28 patients (median age at diagnosis, 7.0 [IQR, 5.0-8.8] years), none were lost to follow-up (Table). Median follow-up was 61 (IQR, 48-81) months.

**Table.** Key Clinical Characteristics of Patients With JDM at Baseline

Characteristic	Value (N = 28)
Sex, No. (%)	
Female	15 (54)
Male	13 (46)
Age, median (IQR), y	7.0 (5.0-8.8)
Time from onset of symptoms to first visit to our department, median (IQR), mo	3 (1-6)
Gottron papule, No. (%)	17 (61)
Heliotrope rash, No. (%)	16 (57)
Muscle weakness, No. (%)	28 (100)
Arthritis or arthralgia, No. (%)	5 (18)
Fever, No. (%)	6 (21)
Severe JDM, No. (%)	7 (25)
Myositis autoantibodies, No./total No. (%) positive	9/13 (69)
MSA (NXP2, TIF1, cN1A, Jo-1, SAE, EJ), No.	8 (2, 2, 1, 1, 1, 1)
MAA (RO-52), No.	1
Muscle enzyme levels, median (IQR), U/L	
Creatine kinase	956.5 (190.8-2222.0)
Aspartate aminotransferase	85.9 (61.1-223.8)
Alanine aminotransferase	48.6 (25.0-138.7)
Lactate dehydrogenase	532 (488.5-774.8)

Abbreviations: JDM, juvenile dermatomyositis; MAA, myositis-associated autoantibody; MSA, myositis-associated autoantibody.

SI conversion factors: To convert creatine kinase, aspartate aminotransferase, alanine aminotransferase, and lactate dehydrogenase to microkatals per liter, multiply by 0.0167.

During IVMP intervals, median oral prednisone dose was 7.5 (IQR, 5.6-10.0) mg. Median time to discontinue GCs was 23.5 (IQR, 21.0-26.0) months and to discontinue all medications was 29.5 (IQR, 27.3-31.8) months.

After treatment initiation, muscle enzymes normalized (median, 3 [IQR, 1-3] months); CMAS scores increased sustainably, with 12-month scores 21.8 (95% CI, 18.2-25.3) points higher than baseline (*P* < .001) (Figure, A). Total DAS decreased, with 12-month scores 10.3 (95% CI, 9.5-11.1) points lower than baseline (*P* < .001) (Figure, B). Twenty-six patients (93%) achieved complete clinical response (median, 10 [IQR, 8.8-18.0] months) and clinical remission (median, 35.5 [IQR, 33.0-37.0] months) (Figure, C). Five patients (18%) relapsed; none had calcinosis or interstitial lung disease.

**Figure.**
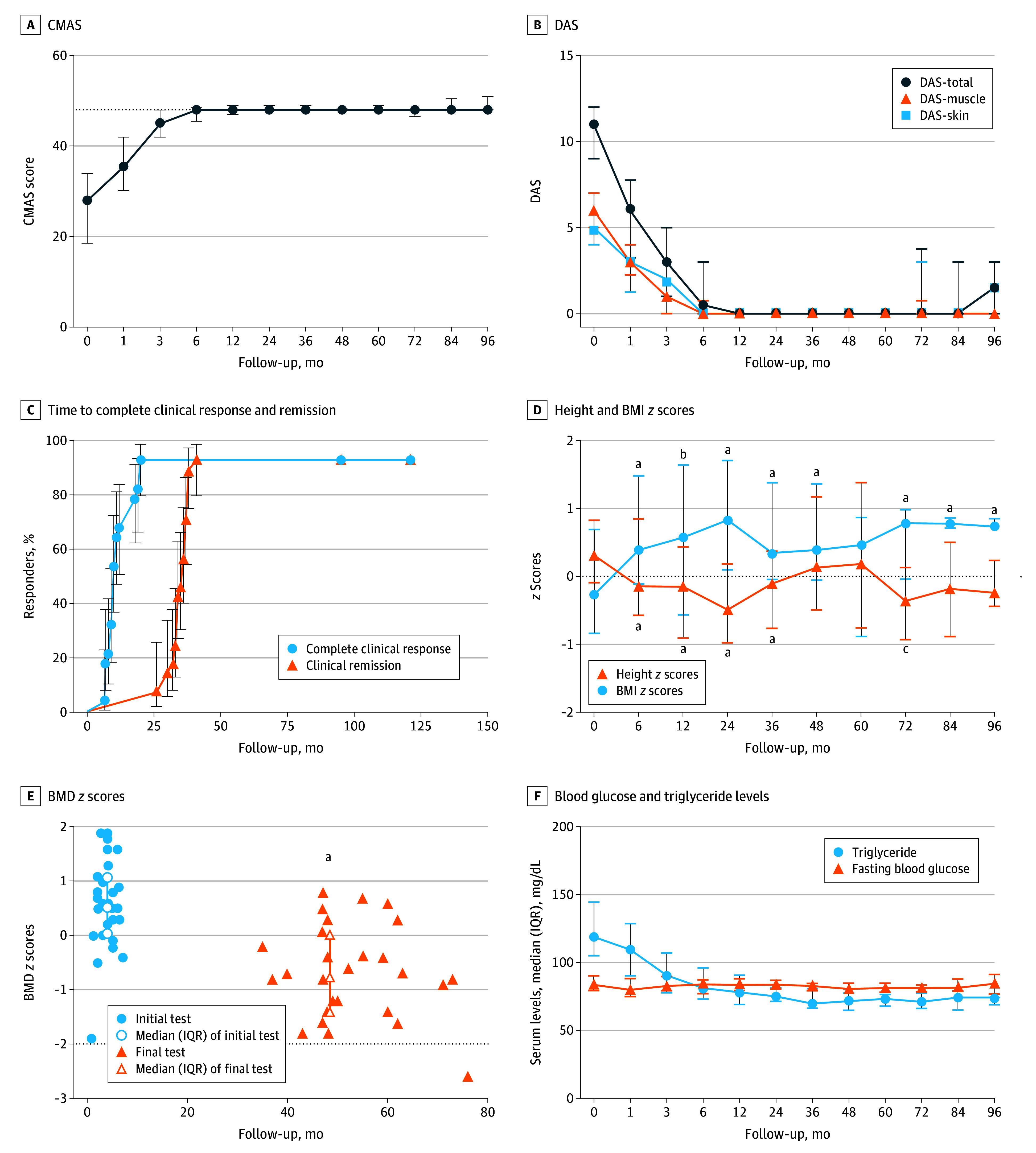
Changes in Disease Activity Score (DAS), Proportions of Complete Clinical Response and Remission, and Safety Indicators A, The change trajectory of Childhood Myositis Assessment Scale (CMAS) scores during follow-up revealed a significant increasing trend (*P* < .001). The dotted line represents the lower bound of the reference limit (CMAS scores = 48). B, The change trajectory of DASs, muscle DASs, and skin DASs during follow-up revealed a significant decreasing trend (*P* < .001). C, Time to complete clinical response and clinical remission. Kaplan-Meier curves show the proportion of patients achieving complete clinical response and clinical remission over time after treatment initiation. D, During follow-up, height *z* scores showed a downward trend initially, then an upward trend, reaching a nadir at 24 months (*P* < .001); body mass index (BMI) *z* scores showed an upward trend initially, then downward, reaching a peak at 24 months (significant difference when compared with baseline [^a^*P* < .001; ^b^*P* < .05; ^c^*P* < .01]). E, Bone mineral density (BMD) *z* scores. BMD *z* score below the dotted line indicates low BMD (significant difference when compared with initial tests [^a^*P* < .001]). F, Serum fasting blood glucose was stable, and triglyceride levels showed a significant increasing trend (*P* < .001). SI conversion factors: To convert glucose to millimoles per liter, multiply by 0.0555; and triglycerides to millimoles per liter, multiply by 0.0113.

Major infections occurred at 2.3 per 100 person-years (no intensive care unit admissions or deaths). Height *z* scores reached a nadir at 24 months (0.7 [95% CI, 0.5-0.9] lower than baseline; *P* < .001) (Figure, D). BMD *z* scores decreased significantly (from 0.6 [IQR, 0.1-1.1] at baseline to −0.8 [IQR, −1.4 to 0.03] at final test [median, 48.5 (IQR, 47.0-60.0) months]; *P* < .001) (Figure, E). Triglyceride levels decreased, with 12-month levels 63.7 (95% CI, 38.1-88.5) mg/dL (to convert millimoles per liter, multiply by 0.0113) lower than baseline (*P* < .001) (Figure, F). Six patients had transient steroid-induced ocular hypertension, 4 with transient glaucoma; 1 had cataracts; and no osteonecrosis or fractures occurred.

## Discussion

Twenty-six patients (93%) achieved complete clinical response and clinical remission. Adverse events were mild and manageable. These findings reflect favorable treatment responses, safety profiles, and good patient tolerance, suggesting this regimen was associated with clinical benefits for JDM. The novelty of this regimen was the use of MMF as first-line therapy. Intermittent IVMP is beneficial for reducing GCs’ adverse effects on growth and development.^1,2^ This study suggests that the regimen, especially the use of MMF, is a therapeutic option for JDM. Limitations include the retrospective design, lack of a control group, small sample size, and use of intravenous immunoglobulin and hydroxychloroquine, which may interfere with interpretation of patients’ treatment responses.

## Notes

Supplement 1. **eFigure.** Schematic Diagram of Intermittent IVMP Therapy
eMethods.

eReferences.

Supplement 2.
Data Sharing Statement

## References

[1] Pan L, Liu J, Liu C, Guo L, Punaro M, Yang S. Childhood-onset systemic lupus erythematosus: characteristics and the prospect of glucocorticoid pulse therapy. Front Immunol. 2023;14:1128754. doi:10.3389/fimmu.2023.1128754 37638017 PMC10448525

[2] Pan L, Liu J, Liu C, Guo L, Yang S. Intermittent pulses of methylprednisolone with low-dose prednisone attenuate lupus symptoms in B6.MRL-Fas^lpr^/J mice with fewer glucocorticoid side effects. Biomed Pharmacother. 2024;177:117138. doi:10.1016/j.biopha.2024.117138 39018878

[3] Hoff LS, de Souza FHC, Miossi R, Shinjo SK. Long-term effects of early pulse methylprednisolone and intravenous immunoglobulin in patients with dermatomyositis and polymyositis. Rheumatology (Oxford). 2022;61(4):1579-1588. doi:10.1093/rheumatology/keab597 34302454

[4] Bhat R, Tonutti A, Timilsina S, Selmi C, Gershwin ME. Perspectives on mycophenolate mofetil in the management of autoimmunity. Clin Rev Allergy Immunol. 2023;65(1):86-100. doi:10.1007/s12016-023-08963-3 37338709

[5] Varnier GC, Consolaro A, Cheng IL, Silva Riveiro A, Pilkington C, Ravelli A. Experience with the use of mycophenolate mofetil in juvenile idiopathic inflammatory myopathies. Rheumatology (Oxford). 2023;62(SI2):SI163-SI169. doi:10.1093/rheumatology/keac404 35929784

