# Supplementary material for: Intermittent Glucocorticoid Pulse Combined With Mycophenolate Mofetil in Juvenile Dermatomyositis
Source: JAMA Dermatol. 2025 Nov 19;162(1):97–9. doi: 10.1001/jamadermatol.2025.4483 (PMC12631562; doi:10.1001/jamadermatol.2025.4483)
Supplement: Supplement 1. — eFigure. Schematic Diagram of Intermittent IVMP Therapy eMethods. eReferences. [file jamadermatol-e254483-s001.pdf]

Supplemental Online Content

Guo L, Liu J, Xin M, et al. Intermittent glucocorticoid pulse combined with mycophenolate mofetil in juvenile dermatomyositis. *JAMA Dermatol*. Published online November 19, 2025. doi:10.1001/jamadermatol.2025.4483

eFigure. Schematic Diagram of Intermittent IVMP Therapy

eMethods.

eReferences.

This supplemental material has been provided by the authors to give readers additional information about their work.

© 2025 Guo L et al. *JAMA Dermatology*.

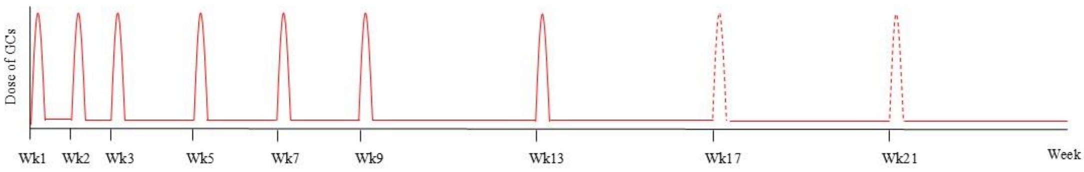

eFigure. Schematic Diagram of Intermittent IVMP Therapy

Each red solid or dotted spike represents a course of IVMP, and one course comprises IVMP (20-30 mg/kg/day, up to 1000 mg) administered once daily for three consecutive days. The red solid spikes indicate that these courses were necessary for all patients, while the red dotted spikes indicate that one or both of these courses might be unnecessary for some patients. One course was administered weekly for the first three courses, every two weeks for the fourth to sixth courses, and every four weeks for the seventh to ninth courses. Oral prednisone 5-10 mg/day was administered during the interval of IVMP and after the discontinuation of pulse therapy. IVMP, intravenous methylprednisolone pulse.

© 2025 Guo L et al. *JAMA Dermatology*.

eMethods.

Exclusion criteria

Patients were excluded if they had other coexisting autoimmune or autoinflammatory diseases, tested positive for the myositis-specific antibody MDA5, or had received GCs therapy for more than one month before initiating treatment at our center.

Definitions

Complete clinical response was defined as no signs of disease activity based on clinical features and laboratory tests for at least 6 consecutive months during treatment[1].

Clinical remission was defined as sustained no disease activity for 6 months after complete discontinuation of all medications[1].

Relapse was defined as clinical or laboratory evidence of reemerging or worsening skin or muscle symptoms requiring adjustment of pharmacological therapy[2].

Severe juvenile dermatomyositis was defined as the presence of intensive care unit treatment requirement, cutaneous ulcers, muscle involvement with a childhood myositis assessment scale score <15, or severe organ involvement (hoarseness and/or dysphagia, cardiac or pulmonary dysfunction, gastrointestinal vasculitis) occurring within the first month of diagnosis[3].

Steroid-induced ocular hypertension was defined as intraocular pressure >21 mmHg in one or both eyes.

Osteoporosis was defined by one or more vertebral compression fractures (regardless of BMD); or ≥2 long-bone fractures by age 10 or ≥3 by 19, plus age/sex-matched BMD/BMC Z-score ≤-2.0 (bone size-corrected)[4].

Major infection was defined as the need for parenteral antimicrobial therapy or oral treatment lasting at least 1 week[5].

Statistical analysis

Statistical analyses were performed using SPSS 20.0 (IBM) and GraphPad Prism 9 (GraphPad Software). Repeated measures were analyzed via generalized estimating equations. Paired comparisons (height/BMI/BMD z-scores vs. baseline) used Wilcoxon signed-rank tests. Time-to-event analyses (complete clinical response and remission) employed Kaplan-Meier methods from treatment initiation, with censoring at last follow-up.

© 2025 Guo L et al. *JAMA Dermatology*.

eReferences.

1. Kishi, T., et al., Corticosteroid discontinuation, complete clinical response and remission in juvenile dermatomyositis. Rheumatology (Oxford). 2021. 60(5): p. 2134-2145.
2. Sun, C., et al., Juvenile dermatomyositis: a 20-year retrospective analysis of treatment and clinical outcomes. Pediatr Neonatol. 2015. 56(1): p. 31-9.
3. Dabbak, I., et al., Efficacy and tolerance of corticosteroids and methotrexate in patients with juvenile dermatomyositis: a retrospective cohort study. Rheumatology (Oxford). 2022. 61(11): p. 4514-4520.
4. Marrani E, Giani T, Simonini G, Cimaz R. Pediatric Osteoporosis: Diagnosis and Treatment Considerations. Drugs. Apr 2017;77(6):679-695. doi:10.1007/s40265-017-0715-3
5. Chen, I.J., et al., Infections in polymyositis and dermatomyositis: analysis of 192 cases. Rheumatology (Oxford). 2010. 49(12): p. 2429-37.

© 2025 Guo L et al. *JAMA Dermatology*.
