# Supplementary material for: Intermittent Glucocorticoid Pulse Combined With Mycophenolate Mofetil in Juvenile Dermatomyositis
Source: JAMA Dermatol. 2025 Nov 19;162(1):97–9. doi: 10.1001/jamadermatol.2025.4483 (PMC12631562; doi:10.1001/jamadermatol.2025.4483)
Supplement: Supplement 2. — Data Sharing Statement [file jamadermatol-e254483-s002.pdf]

Data Sharing Statement

Guo. Intermittent Glucocorticoid Pulse Combined With Mycophenolate Mofetil in Juvenile Dermatomyositis. *JAMA Dermatol*. Published November 19, 2025.
doi:10.1001/jamadermatol.2025.4483

Data
Data available: Yes
Data types: Deidentified participant data
How to access data: Data are available upon reasonable request.Contact email: sryang@jlu.edu.cn
When available: With publication

Supporting Documents
Document types: None

Additional Information
Who can access the data: Researchers whose proposed use of the data has been approved
Types of analyses: For a specified purpose
Mechanisms of data availability: With a signed data access agreement
